# Transmission of Guanarito and Pirital Viruses among Wild Rodents, Venezuela

**DOI:** 10.3201/eid1712.110393

**Published:** 2011-12

**Authors:** Mary L. Milazzo, Maria N.B. Cajimat, Gloria Duno, Freddy Duno, Antonio Utrera, Charles F. Fulhorst

**Affiliations:** University of Texas Medical Branch, Galveston, Texas, USA (M.L. Milazzo, M.N.B. Cajimat, C.F. Fulhorst);; Ministerio de Sanidad y Asistencia Social, Region Sanitaria del Estado Portuguesa, Guanare, Venezuela (G. Duno, F. Duno);; Universidad Nacional Experimental de Los Llanos Occidentales “Ezequiel Zamora,” Guanare (A. Utrera)

**Keywords:** viruses, Guanarito virus, Pirital virus Arenaviridae, Venezuelan hemorrhagic fever, Sigmodon alstoni, Zygodontmys brevicauda, rodents, cane mice, cotton rats, zoonoses, research

## Abstract

Samples from rodents captured on a farm in Venezuela in February 1997 were tested for arenavirus, antibody against Guanarito virus (GTOV), and antibody against Pirital virus (PIRV). Thirty-one (48.4%) of 64 short-tailed cane mice (*Zygodontomys brevicauda*) were infected with GTOV, 1 Alston’s cotton rat (*Sigmodon alstoni*) was infected with GTOV, and 36 (64.3%) of 56 other Alston’s cotton rats were infected with PIRV. The results of analyses of field and laboratory data suggested that horizontal transmission is the dominant mode of GTOV transmission in *Z. brevicauda* mice and that vertical transmission is an important mode of PIRV transmission in *S. alstoni* rats. The results also suggested that bodily secretions and excretions from most GTOV-infected short-tailed cane mice and most PIRV-infected Alston’s cotton rats may transmit the viruses to humans.

## Abstract 2

Secretions and excretions from virus-infected cane mice and cotton rats might transmit disease to humans.

The Tacaribe serocomplex viruses (family *Arenaviridae*, genus *Arenavirus*) known to occur in Venezuela are Guanarito virus (GTOV) and Pirital virus (PIRV) (*1**,**2*). GTOV is the etiologic agent of Venezuelan hemorrhagic fever (VHF) (*1*). The human health significance of PIRV has not been rigorously investigated (*3*).

Specific members of the rodent family Cricetidae (*4*) are the principal hosts of the Tacaribe complex viruses for which natural host relationships have been well characterized. It is generally accepted that humans usually become infected with arenaviruses by inhalation of virus in aerosolized droplets of saliva, respiratory secretions, urine, or blood from infected rodents or by inhalation of virus-contaminated dust particles.

The results of published studies (*2**,**5*) indicated that the short-tailed cane mouse (*Zygodontomys brevicauda*) is the principal host of GTOV and that the Alston’s cotton rat (*Sigmodon alstoni*) is the principal host of PIRV. The objective of our study was to extend knowledge of the natural host relationships of these arenaviruses, particularly the relative importance of various modes of intraspecies virus transmission and the prevalence of virus shedding among naturally infected rodents.

## Materials and Methods

This report is the sixth in a series of publications that include rodents captured in Venezuela in February 1997. The rodents were trapped on Hato Maporal, a farm near the town of Caño Delgadito in the municipality of Guanarito, Portuguesa State (*6*).

### Study Sites

Rodents were trapped at 3 sites on Hato Maporal, designated A, B, and C. Traps were set at site A in tall grassy areas on the edge of a grove of trees, site B in tall grassy areas alongside an unpaved road and in a field filled with weeds, and site C in a field filled with crop stubble and in grassy areas alongside the field. Site A was 0.2 km from site B, and site C was 0.7 km from sites A and B.

### Capture and Processing of Rodents

The rodents were captured in aluminum live-capture traps (*6*). The traps were placed at 8-m intervals, baited with small pieces of freshly cut pineapple, set 1 h before sunset, and checked at daybreak the following day. Each rodent was assigned a unique identification (FHV, Fiebre Hemorrágica Venezolana) number and then killed by exposure to a lethal dose of vaporized chloroform. The identification number, date of capture, trap site and trap number, species identity, sex, total length (tip of nose to tip of tail), length of tail, and other information were recorded on a standardized form. A throat swab; samples of blood, lung, spleen, liver, and kidney; and a sample of urine were collected from each rodent (*6*). These samples were stored in cryo-vials in liquid nitrogen in the field and then shipped on dry ice to the University of Texas Medical Branch (Galveston, TX, USA).

### Virus Assay

The samples from the throat swabs; crude 10% wt/vol homogenates of the samples of lung, spleen, and kidney in 0.01 mol/L phosphate-buffered saline; and samples of urine were assayed for arenavirus by cultivation in monolayers of Vero E6 cells (*5*). Cells harvested from the monolayers on day 13 or 14 postinoculation were tested for arenaviral antigen by using an indirect fluorescent antibody test (IFAT) in which the primary antibody was a mixture of a hyperimmune mouse ascitic fluid (HMAF) raised against the GTOV prototype strain INH-95551 (*7*) and an HMAF raised against the PIRV prototype strain VAV-488 (*2*).

### Serologic Characterization of Viruses

Strains of GTOV were distinguished from strains of PIRV by an ELISA (*5*). The test antigens were detergent lysates of infected Vero E6 cells. Serial 2-fold dilutions (from 1:800 through 1:204,800 vol/vol) of an anti-GTOV HMAF and anti-PIRV HMAF were tested against each antigen. Antibody (IgG) bound to antigen was detected by using a goat antimouse (*Mus musculus*) IgG peroxidase conjugate in conjunction with the ABTS Microwell Peroxidase Substrate System (Kirkegaard and Perry Laboratories, Gaithersburg, MD, USA). The reactivity of an HMAF against an antigen was the sum of the optical densities of the 8 reactions in the series of 4-fold dilutions of the HMAF tested against the antigen. The identity of an isolate was determined by direct comparison of the reactivity of the anti-GTOV HMAF versus the reactivity of the anti-PIRV HMAF against the test antigen.

### Genetic Characterization of Viruses

The sequences of a 616–619-nt fragment of the nucleocapsid (N) protein genes of the arenaviruses isolated from the spleens of 21 rodents in this study (Table 1) were determined to assess the accuracy of the interpretation of the ELISA data. Total RNA was isolated from monolayers of infected Vero E6 cells by using TRIzol Reagent (Invitrogen Life Technologies, Inc., Carlsbad, CA, USA) or Tri Reagent (Sigma Aldrich, St. Louis, MO, USA). First-strand cDNA was synthesized by using SuperScript II RNase H^-^ Reverse Transcriptase (Invitrogen Life Technologies, Inc.) in conjunction with oligonucleotide 19C-cons (*8*). Amplicons were synthesized from first-strand cDNA by using the MasterTaq Kit (Eppendorf North America, Inc., Westbury, NY, USA) in conjunction with oligonucleotides that flank either a 619-nt fragment of the N protein gene of GTOV strain INH-95551 or the homologous region (a 616-nt fragment) of the N protein gene of PIRV strain VAV-488. Amplicons of the expected size were sequenced directly by using the BigDye Terminator v3.1 Cycle Sequencing Kit (Applied Biosystems, Inc., Foster City, CA, USA).

**Table 1:** Strains of Guanarito virus and Pirital virus isolated from the spleens of rodents captured on Hato Maporal and included in the analysis of nucleocapsid protein gene sequences, municipality ofGuanarito, Portuguesa State, Venezuela, 1994–1997

Virus and strain*	Rodent no.†	Species	Date captured	Trap site	GenBank accession no.
Guanarito					
VAV-623	FHV-623	*Zygodontomys brevicauda*	1994 Mar 23	–	JF412365
VAV-952	FHV-952	*Z. brevicauda*	1994 Jun 6	–	JF412366
AV 97020997	FHV-4030	*Z. brevicauda*	1997 Feb 4	A	JF412369
AV 97021004	FHV-4037	*Z. brevicauda*	1997 Feb 4	A	JF412370
AV 97021033	FHV-4066	*Z. brevicauda*	1997 Feb 5	A	JF412371
AV 97021034	FHV-4067	*Z. brevicauda*	1997 Feb 5	A	JF412372
AV 97021073	FHV-4106	*Z. brevicauda*	1997 Feb 11	B	JF412373
AV 97021084	FHV-4117	*Z. brevicauda*	1997 Feb 11	B	JF412374
AV 97021092	FHV-4125	*Z. brevicauda*	1997 Feb 11	B	JF412375
AV 97021104	FHV-4137	*Z. brevicauda*	1997 Feb 12	B	JF412376
AV 97021106	FHV-4139	*Z. brevicauda*	1997 Feb 12	B	JF412377
AV 97021113	FHV-4146	*Z. brevicauda*	1997 Feb 12	B	JF412378
AV 97021116	FHV-4149	*Sigmodon alstoni*	1997 Feb 12	C	JF412379
AV 97021117	FHV-4150	*Z. brevicauda*	1997 Feb 12	C	JF412380
AV 97021119	FHV-4152	*Z. brevicauda*	1997 Feb 12	C	AY573922
Pirital					
VAV-628	FHV-628	*S. alstoni*	1994 Mar 23	–	JF412367
VAV-956	FHV-956	*Z. brevicauda*	1994 Jun 19	–	JF412368
AV 97021016	FHV-4049	*S. alstoni*	1997 Feb 4	B	AY573923
AV 97021026	FHV-4059	*S. alstoni*	1997 Feb 4	B	JF412381
AV 97021027	FHV-4060	*S. alstoni*	1997 Feb 4	B	JF412382
AV 97021028	FHV-4061	*S. alstoni*	1997 Feb 4	B	JF412383
AV 97021029	FHV-4062	*S. alstoni*	1997 Feb 4	B	JF412384
AV 97021030	FHV-4063	*S. alstoni*	1997 Feb 5	A	JF412385
AV 97021036	FHV-4069	*S. alstoni*	1997 Feb 5	A	JF412386
AV 97021040	FHV-4073	*S. alstoni*	1997 Feb 5	A	JF412387
AV 97021112	FHV-4145	*S. alstoni*	1997 Feb 12	B	JF412388
AV 97021120	FHV-4153	*S. alstoni*	1997 Feb 12	C	JF412389

*Strains VAV-623, VAV-628, VAV-952, VAV-956, AV 97021016, and AV 97021119 were published previously (*3**,**5*). The nucleotide sequences of the 21 other strains were determined in this study. –,rodents were captured in 1994 before establishment of trap sites. †FHV, Fiebre Hemorrágica Venezolana.

### Antibody Assay

Blood samples were rendered noninfectious by irradiation (5 × 10^6^ rads, Co^60^ source), diluted 1:20 vol/vol in phosphate-buffered saline, and then tested for IgG against GTOV strain INH-95551 and PIRV strain VAV-488 by using an IFAT. The cell spots were either a mixture of Vero E6 cells infected with INH-95551 and uninfected Vero E6 cells or a mixture of Vero E6 cells infected with VAV-488 and uninfected Vero E6 cells. Antibody bound to antigen was revealed by using a fluorescein isothiocyanate–conjugated goat antibody raised against mouse (*M. musculus*) IgG (Kirkegaard and Perry). End-point titers against INH-95551 and VAV-488 were measured in the positive samples by using serial 2-fold dilutions beginning at 1:20 and ending at 1:640 vol/vol.

### Data Analysis

The male short-tailed cane mice, female short-tailed cane mice, male Alston’s cotton rats, and female Alston’s cotton rats were assigned on the basis of their nose-to-rump lengths (measured in mm) to 4 size categories. In each instance, the upper boundary of class I was the mean length − 1 SD, the upper boundary of class II was the mean length, the upper boundary of class III was the mean length + 1 SD, and the upper boundary of class IV was the longest nose-to-rump length (Table 2). Animals that were culture-positive or antibody-positive were treated as infected. The acceptable type I error in all statistical tests was α = 0.05.

**Table 2:** Prevalence of Guanarito virus infection in short-tailed cane mice (*Zygodontomys brevicauda*) and Pirital virus infection in Alston’s cotton rats (*Sigmodon alstoni*) captured on Hato Maporal, municipality of Guanarito, Portuguesa State, Venezuela, February 1997

Size class*	Short-tailed cane mice, no. infected/no. tested†				Alston’s cotton rats, no. infected/no. tested‡		
M	F	Total	M	F	Total
I	1/5	0/2	1/7		5/6	3/3	8/9
II	2/8	5/15	7/23		6/8	3/8	9/16
III	9/13	7/13	16/26		6/11	7/9	13/20
IV	2/3	5/5	7/8		3/7	3/4	6/11
Total	14/29	17/35	31/64		20/32	16/24	36/56

*The boundaries of the size classes were based on analyses of nose-to-rump lengths (measured in mm). Male cane mice: I, 75.0–100.9; II, 101.0–117.1; III, 117.2–133.4; IV, 133.5–147.0. Female cane mice: I, 64.0–99.0; II, 99.1–111.2; III, 111.3–123.4; IV, 123.5–135.0. Male cotton rats: I, 89.0–105.6; II, 105.6–123.6; III, 123.6–141.6; IV, 141.6–150.0. Female cotton rats: I, 83.0–100.8; II, 100.8–119.6; III, 119.6–138.4; IV, 138.4–146.0. †Guanarito virus (GTOV) was isolated from 29 cane mice. Antibody against GTOV was found in 11 of the 29 culture-positive cane mice and 2 of the 35 culture-negative cane mice. ‡Pirital virus (PIRV) was isolated from all of the infected cotton rats, and antibody against PIRV was found in 1 of the culture-positive cotton rats. The table does not include FHV-4149, the only cotton rat infected with GTOV.

The analyses of the nucleotide sequences included GTOV strain INH-95551 (GenBank accession no. U43686); GTOV strains VAV-623, VAV-952, and AV 97021119 (Table 1); PIRV strain VAV-488 (GenBank accession no. U62561); and PIRV strains VAV-628, VAV-956, and AV 97021016 (Table 1). Strain INH-95551 was isolated from a patient who died of VHF (*7*); VAV-488 was isolated from an Alston’s cotton rat captured ≈54 km east-northeast of Hato Maporal (*2*); VAV-623, VAV-628, VAV-952, and VAV-956 were isolated from rodents captured on Hato Maporal in 1994 (*5*); and AV 97021016 and AV 97021119 were isolated from rodents captured on Hato Maporal in 1997 and reported previously (*3*). The neighbor-joining analysis of genetic (p) distances was done with MEGA version 4.0 (*9*). Bootstrap support (*10*) for the results of the neighbor-joining analysis was based on 1,000 pseudoreplicate datasets generated from the original multiple nucleotide sequence alignment.

Antibody titers <20 were considered 10 in comparisons of antibody titers to GTOV strain INH-95551 and PIRV strain VAV-488 in individual blood samples. The apparent homologous virus in an antibody-positive sample was the virus associated with the highest titer if the absolute value of the difference between the titers to GTOV and PIRV was >4-fold.

## Results

A total of 128 rodents were captured on Hato Maporal in February 1997 in 1,000 trap-nights, with an overall trap success rate of 12.8% (Table 3). Most (121 [94.5%]) of the 128 rodents were short-tailed cane mice or Alston’s cotton rats.

**Table 3:** Prevalence of arenavirus infections in rodents captured on Hato Maporal, municipality of Guanarito, Portuguesa State, Venezuela, February 1997

Site*	No. traps	No. trap-nights	Trap success rate (%)	Prevalence of infection†			Total rodents
				Cotton rat	Cane mouse	Rice rat	
A	80	160	36/160 (22.5)	5/14	12/20	0/2	17/36
B	160	640	82/640 (12.8)	30/41	15/37	0/4	45/82
C	100	200	10/200 (5.0)	2/2	4/7	0/1	6/10
Total	340	1,000	128/1,000 (12.8)	37/57	31/64	0/7	68/128

*Traps were set at site A on February 4 and 5; site B on February 4, 5, 11, and 12; and site C on February 5 and 12. †Number of infected (culture-positive or antibody-positive) rodents/total number of rodents tested (captured). Cotton rat, Alston’s cotton rat (*Sigmodon alstoni*); cane mouse, short-tailed cane mouse (*Zygodontomys brevicauda*); rice rat, delicate pygmy rice rat (*Oligoryzomys delicatus*).

Fifty-seven (89.1%) of the 64 short-tailed cane mice and 55 (96.5%) of the 57 Alston’s cotton rats were captured in 91 (37.9%) of the 240 traps set on sites A and B. Six Alston’s cotton rats from site A were found in traps adjacent to traps in which cane mice were captured, 17 Alston’s cotton rats from site B were found in traps adjacent to traps in which cane mice were captured, and a cotton rat and cane mouse were captured on different nights in each of 3 traps on site A and 6 traps on site B. Collectively, these observations suggest that the short-tailed cane mice captured on sites A and B lived in close proximity to Alston’s cotton rats and vice versa.

Arenavirus was isolated from the throat swabs; samples of lung, spleen, or kidney; and samples of urine from 29 (45.3%) of the 64 short-tailed cane mice, 37 (64.9%) of the 57 Alston’s cotton rats, and none of the 7 pygmy rice rats (Table 4). The analyses of the ELISA data indicated that the arenaviruses isolated from Alston’s cotton rat FHV-4149 and the short-tailed cane mice are strains of GTOV and that the arenaviruses isolated from the Alston’s cotton rats other than FHV-4149 are strains of PIRV. The results of the neighbor-joining analysis of N protein gene sequence data (Figure) were 100% concordant with the serologic identities of the 21 viruses selected for genetic characterization, GTOV strain AV 97021119, and PIRV strain AV 97021016.

**Table 4:** Isolation of arenaviruses from 64 short-tailed cane mice (*Zygodontomys brevicauda)* and 57 Alston’s cotton rats (*Sigmodon alstoni)* captured on Hato Maporal, municipality of Guanarito, Portuguesa State, Venezuela, February 1997

No.	Sample*					Antibody status
	Throat swab	Lung	Spleen	Kidney	Urine	
Cane mice†						
3	–	+	–	–	–	–
5	–	–	+	–	–	–
6	–	+	+	+	+	–
	–	–	+	+	+	–
1	+	–	+	+	+	–
2	+	+	+	+	+	–
2	+	+	+	+	+	+
2	+	–	+	+	+	+
6	–	–	+	+	+	+
1	–	–	+	+	–	+
2	–	–	–	–	–	+
33	–	–	–	–	–	–
Cotton rats‡						
2	–	–	+	–	–	–
4	–	+	+	+	–	–
21	–	+	+	+	+	–
9	+	+	+	+	+	–
1	–	–	+	–	–	+
20	–	–	–	–	–	–

*–, negative; +, positive. †The arenaviruses isolated from the cane mice were strains of Guanarito virus. ‡The arenavirus isolated from cotton rat FHV-4149 was a strain of Guanarito virus. The arenaviruses isolated from the 36 other culture-positive cotton rats were strains of Pirital virus.

**Figure:**
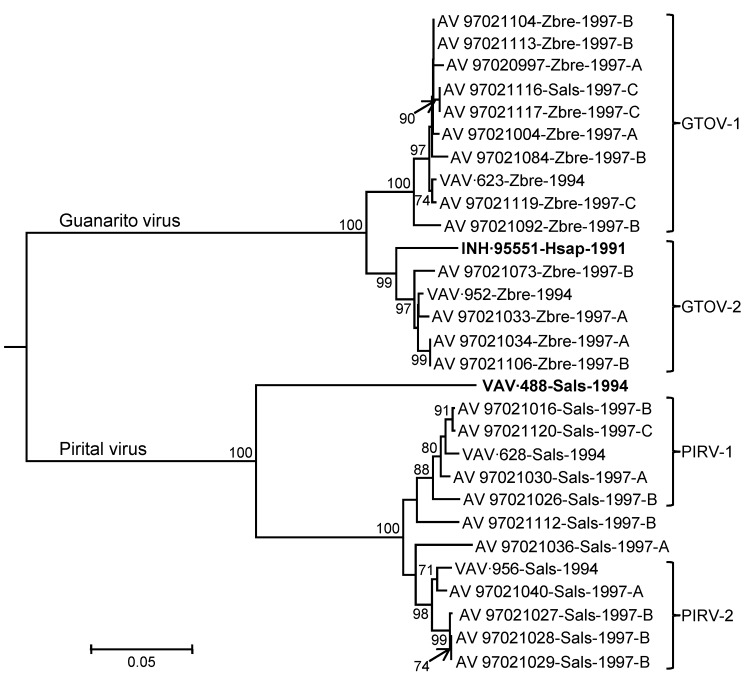
Phylogenetic relationships among 27 arenaviruses isolated from rodents captured on Hato Maporal, Portuguesa State, Venezuela, 1994 or 1997; Guanarito virus (GTOV) prototype strain INH-95551 (**boldface**); and Pirital virus (PIRV) prototype strain VAV-488 (**boldface**) based on a neighbor-joining analysis of nucleocapsid protein gene sequence data. Branch lengths are proportional to genetic (p) distances; the numbers at the nodes indicate the percentage of 1,000 bootstrap replicates that supported the interior branches; bootstrap support values <70% are not listed; and the analysis was rooted to Oliveros virus strain 3229–1 (GenBank accession no. NC_010248). The branch labels include (in the following order) virus strain, host species, year of isolation, and (viruses from 1997) the study site at which the infected rodent was captured. Hsap, *Homo sapiens*; Sals, *Sigmodon alstoni*; Zbre, *Zygodontomys brevicauda*.

Nucleotide sequence nonidentity between the GTOV strains from 1994 (i.e., VAV-623 and VAV-952) was 6.0%, nucleotide sequence nonidentities among AV 97021119 and the 12 other GTOV strains from 1997 ranged from 0 to 7.3%, and nucleotide sequence nonidentities between the GTOV strains from 1994 and the 13 GTOV strains from 1997 ranged from 0.3% to 6.6%. Similarly, nucleotide sequence nonidentity between the PIRV strains from 1994 (i.e., VAV-628 and VAV-956) was 5.2%, nucleotide sequence nonidentities among AV 97021016 and the 9 other PIRV strains from 1997 ranged from 0 to 6.3%, and nucleotide sequence nonidentities between the PIRV strains from 1994 and the 10 PIRV strains from 1997 ranged from 1.0% to 6.3%.

Antibody (IgG) against GTOV or PIRV was found in 11 (37.9%) of the 29 culture-positive cane mice, 2 (5.7%) of the 35 culture-negative cane mice, 1 (2.7%) of the 37 culture-positive cotton rats, none of the 20 culture-negative cotton rats, and none of the 7 pygmy rice rats (Table 4). The only antibody-positive cotton rat (FHV-4124) was mature (size class III) and antibody positive to GTOV and PIRV. None of the 7 cane mice in size class I was antibody positive to GTOV or PIRV.

The end-point antibody titers to GTOV in the antibody-positive cane mice ranged from 40 to 160, none of the cane mice were antibody positive to PIRV, and the end-point antibody titers to GTOV and PIRV in the antibody-positive Alston’s cotton rat were 40 and >640, respectively. Thus, GTOV was the apparent homologous virus in all of the antibody-positive cane mice, and PIRV was the apparent homologous virus in the antibody-positive cotton rat.

Eight (26.7%) of the 30 short-tailed cane mice in size classes I and II, 23 (67.6%) of the 34 cane mice in size classes III and IV, 11 (68.8%) of the 16 male short-tailed cane mice in size classes III and IV, and 12 (66.7%) of the 18 female short-tailed cane mice in size classes III and IV were infected with GTOV (Table 2). The prevalence of infection in the 34 short-tailed cane mice in size classes III and IV differed significantly from that in the 30 cane mice in size classes I and II (2-tailed Fisher exact test p<0.01), but the prevalence of infection in the 16 male short-tailed cane mice in size classes III and IV did not differ significantly from that in the 18 female short-tailed cane mice in size classes III and IV (2-tailed Fisher exact test p = 0.72).

Thirteen (86.7%) of the 15 female short-tailed cane mice in size class II, 7 (53.8%) of the 13 female short-tailed cane mice in size class III, and 5 (100%) of the 5 female short-tailed cane mice in size class IV were pregnant. Furthermore, 4 (30.8%) of the 13 pregnant short-tailed cane mice in size class II, 5 (71.4%) of the 7 pregnant short-tailed cane mice in size class III, and all 5 of the pregnant short-tailed cane mice in size class IV were infected with GTOV.

As indicated previously, 12 (66.7%) of the 18 female short-tailed cane mice in size classes III and IV were infected with GTOV. Yet only 1 (14.3%) of the 7 short-tailed cane mice in size class I was infected with GTOV (Table 2). The difference between the prevalence of infection in the female short-tailed cane mice in size classes III and IV and that in the short-tailed cane mice in size class I was significant (2-tailed Fisher exact test p = 0.03).

Seventeen (65.4%) of the 26 Alston’s cotton rats in size classes I and II, 19 (61.3%) of the 31 Alston’s cotton rats in size classes III and IV, 9 (50.0%) of the 18 male Alston’s cotton rats in size classes III and IV, and 10 (76.9%) of the 13 female Alston’s cotton rats in size classes III and IV were infected with PIRV (Table 2). The prevalence of PIRV infection in the Alston’s cotton rats in size classes III and IV did not differ significantly from that in the Alston’s cotton rats in size classes I and II (2-tailed Fisher exact test p = 0.78), and the prevalence of infection in the male Alston’s cotton rats in size classes III and IV did not differ significantly from that in the female Alston’s cotton rats in size classes III and IV (2-tailed Fisher exact test p = 0.16).

## Discussion

The results of this study affirm conclusions drawn from previous studies (*2**,**5*). Specifically, the short-tailed cane mouse is the principal host of GTOV, and Alston’s cotton rat is the principal host of PIRV. Examples of GTOV infection in rodents other than the short-tailed cane mouse are limited to the isolation of AV 97021116 from Alston’s cotton rat FHV-4149 and the isolation of GTOV from a pygmy rice rat (*Oligoryzomys* sp.) and 4 Alston’s cotton rats captured at localities in Venezuela other than Hato Maporal (*11*). Similarly, examples of PIRV infection in rodents other than Alston’s cotton rats are limited to the isolation of VAV-956 from a short-tailed cane mouse captured on Hato Maporal in 1994 (*5*) and the isolation of PIRV from 5 short-tailed cane mice and a spiny rat (*Proechimys* sp.) captured at other localities in Venezuela (*12*).

The results of the analysis of the capture data in this study suggest that the short-tailed cane mice captured on sites A and B lived in close physical association with Alton’s cotton rats and vice versa. Yet none of the 57 short-tailed cane mice captured on A or B were infected with PIRV, and only 1 of the 55 Alston’s cotton rats captured on A or B was infected with GTOV. Collectively, these observations suggest that intimate social interactions between short-tailed cane mice and Alston’s cotton rats are infrequent. Alternatively, GTOV-infected short-tailed cane mice are rarely infectious to Alston’s cotton rats and PIRV-infected Alston’s cotton rats are rarely infectious to short-tailed cane mice.

Chronic infections in individual rodents appear to be critical to the long-term maintenance of arenaviruses in nature. Factors that likely affect the duration of GTOV infection in naturally infected short-tailed cane mice include age at exposure to GTOV, host genetics, virus genetics, inoculum dose, and route of exposure (*13**,**14*).

The positive association between prevalence of infection and size class in the short-tailed cane mice suggests that most GTOV infections in short-tailed cane mice are acquired in an age-dependent manner. Allogrooming, mating, intraspecies aggression, and other activities that entail close physical contact may facilitate horizontal transmission in *Z. brevicauda* mice. The isolation of GTOV from the samples of lung but not the samples of spleen or kidney from 3 antibody-negative short-tailed cane mice (Table 4) suggests that these animals were infected by way of the respiratory tract rather than by wounding or venereal contact. The lack of an association between prevalence of infection and sex in the short-tailed cane mice in size classes III and IV suggests that male animals and female animals contribute equally to the transmission of GTOV in *Z. brevicauda* mice.

Under the assumption that short-tailed cane mice whelp their first offspring after they reach size class III, the high prevalence of infection in the female cane mice in size classes III and IV together with the low prevalence of infection in the cane mice in size class I suggest that vertical (dam-to-progeny) transmission of GTOV in *Z. brevicauda* mice is uncommon. Perhaps GTOV infection in the cane mouse fetus is lethal late in gestation. Alternatively, the survivorship of congenitally infected short-tailed cane mice may be significantly less than the survivorship of their uninfected counterparts during birth through weaning.

Together, the high prevalence of PIRV infection in the Alston’s cotton rats in size class 1 and the lack of an association between prevalence of infection and size class in the Alston’s cotton rats suggest that most cotton rats become infected with PIRV at an early age, perhaps in utero or immediately postpartum. Hypothetically, vertical (dam-to-progeny) virus transmission is the dominant mode of PIRV transmission in *S. alstoni* rats.

Arenavirus was isolated from the throat swabs and/or samples of urine from 20 (64.5%) of the 31 infected short-tailed cane mice and 30 (83.3%) of the 36 PIRV-infected Alston’s cotton rats in this study (Table 4), suggesting that bodily secretions or excretions from most GTOV-infected short-tailed cane mice and most PIRV-infected Alston’s cotton rats can transmit the viruses to humans. In a laboratory study (*13*), newborn, juvenile, and some adult short-tailed cane mice inoculated with GTOV strain INH-95551 persistently shed virus in saliva, respiratory secretions, or urine through day 208 postinoculation. Whether the magnitude and duration of virus shedding in PIRV-infected Alston’s cotton rats are comparable with that in GTOV-infected short-tailed cane mice has not been investigated.

VHF was first recognized as a distinct clinical entity during an outbreak of hemorrhagic fever that began in 1989 in Guanarito (*1*). From September 1989 through December 2006, the State of Portuguesa recorded 618 VHF cases, with a case-fatality rate of 23.1% (*3*). Most (610 [98.7%]) patients lived or worked in rural areas in Guanarito when they became ill with VHF.

GTOV is presumed to be the only agent of VHF; however, the majority of the arenaviruses isolated from VHF patients during September 1989 through December 2006 were identified as strains of GTOV solely on the basis of the results of an IFAT in which extensive cross-reactivity between PIRV and GTOV is possible (*5*). Thus, the arenaviruses isolated from some VHF cases may be strains of PIRV.

PIRV, in association with *S. alstoni* rats, is widely distributed in rural areas in Guanarito and elsewhere in Portuguesa State (*2**,**5**,**12*). Furthermore, Alston’s cotton rats (like short-tailed cane mice) are common in grass-dominated habitats, for example, tall grass and hedgerows adjacent to cultivated fields and areas with tall grass alongside human dwellings. Thus, the epidemiology of PIRV infection likely is highly similar to the epidemiology of GTOV infection in Portuguesa, with most infections in persons who live or work in rural areas in Guanarito.

The neighbor-joining analysis of N protein sequence data separated the viruses from Hato Maporal into 4 groups: GTOV-1, GTOV-2, PIRV-1, and PIRV-2 (Figure). Each group included a strain from 1994 and strains from 1997, suggesting that multiple evolutionary lineages of GTOV and multiple evolutionary lineages of PIRV were maintained on Hato Maporal during mid-June 1994 through early February 1997. Whether GTOV-1 viruses differ from GTOV-2 viruses with regard to pathogenicity in humans or cane mice and whether PIRV-1 viruses differ from PIRV-2 viruses with regard to pathogenicity in humans or cotton rats has not been investigated.
